# Timed 360° turn test in people with Alzheimer′s disease: a reliability and validity study

**DOI:** 10.1007/s11845-025-04258-y

**Published:** 2026-01-13

**Authors:** Fatih Soke, Nigar Esra Erkoc Ataoglu, Cagri Gulsen, Duygu Korkem Yorulmaz, Fatma Nurveren Gulfirat, Hatice Ayse Tokcaer Bora

**Affiliations:** 1 Department of Physiotherapy and Rehabilitation, University of Health Sciences, Gulhane Faculty of Physiotherapy and Rehabilitation, Ankara, Turkey; 2 Department of Neurology, Gazi University, Faculty of Medicine, Ankara, Turkey; 3 Department of Physiotherapy and Rehabilitation, Osmangazi University, Faculty of Health Sciences, Eskisehir, Turkey; 4 Department of Therapy and Rehabilitation, Ankara University, Kizilcahamam Vocational School of Health Service, Ankara, Turkey

**Keywords:** Alzheimer’s disease, Outcome measures, Rehabilitation, Reliability, Timed 360 º turn test, Validity

## Abstract

**Background:**

Turning is an essential and challenging activity in daily life but is not specifically assessed for people with Alzheimer’s disease (PwAD). The timed 360º turn test (360TT) is a specific tool assessing turning ability; however, its reliability and validity have not been established in Alzheimer’s disease (AD).

**Aims:**

To investigate: (1) the test-retest reliability of the 360TT in PwAD; (2) the standard error of measurement (SEM) and minimum detectable change (MDC) in the 360TT times; (3) the concurrent and known-groups validity of the 360TT times.

**Methods:**

This cross-sectional study included 33 PwAD and 32 healthy people. The 360TT was administered along with the Berg Balance Scale, Timed Up and Go Test, and Mini-Mental State Examination. The test-retest reliability of the 360TT was examined by performing it twice at a 7–10 day interval for PwAD.

**Results:**

Test-retest reliability of the 360TT was excellent for the dominant and non-dominant sides (ICC = 0.957 and ICC = 0.916, respectively). The SEM_95_ and MDC_95_ values were 0.33 s and 0.91 s for the dominant side, while these values were 0.31 s and 0.85 s for the non-dominant side. The 360TT was correlated with the BBS, TUG, and MMSE in both sides (*p* < 0.05). PwAD took longer to complete the 360TT on both sides compared to healthy people (*p* < 0.001).

**Conclusions:**

The 360TT is a reliable and valid method in the evaluation of turning ability for PwAD. Clinicians and researchers can also use the 360TT to quantify changes in turning ability in AD.

## Introduction

Alzheimer’s disease (AD), the most common neurodegenerative disease, is characterized by a progressive decline in cognitive function [1]. Although cognitive disorders such as cognitive dysfunction, memory loss, disorientation, and learning difficulties are the most commonly and well-documented symptoms and signs of the disease, people with AD (PwAD) typically experience motor impairments as well [2]. The common motor deficits are gait disturbances [3], balance deficits, mobility problems, and increased risk of falls [4]. These impairments deteriorate motor performance and contribute to functional decline in PwAD [5].

Turning is an essential component of mobility and accounts for over 40% of the steps taken in daily life. Individuals require some amount of turning in nearly every functional task performed throughout the day [6]. Turning requires greater neural resources, stronger coordination between balance and gait systems, and more precise spatial control of the limbs, making it more challenging and complex than linear gait [7, 8]. However, turning is not specifically assessed in rehabilitation and is only one component of commonly used balance and mobility assessments for PwAD. For example, clinicians can assess turning performance by administering the Berg Balance Scale (BBS) including a 360° turn [9], or the timed up and go test (TUG) including a 180° turn [10]. Given the importance of turning and a lack of specific method for measuring turning performance, the assessment of turning is a crucial component of clinical research and practice in AD.

The timed 360° turn test (360TT) was originally developed to assess turning performance. It is a simple, and time-efficient test. Individuals are required to perform a full rotation around their vertical axis to complete the test [11]. The 360TT has excellent test-retest reliability in other neurological disorders such as stroke [11], and Parkinson’s disease [12] (intraclass correlation coefficient [ICC] = 0.824–0.951, and ICC = 0.860–0.932, respectively). Older adults who take longer than 3.8 s to complete a 360° turn are at significantly increased risk for functional dependence [13]. The 360TT has been correlated with disease-specific impairments such as balance and mobility in stroke [11] and PD [12]. To date, no study has comprehensively investigated the reliability and validity of the 360TT for PwAD. The aims of this study were, therefore, to investigate: (1) the test-retest reliability of the 360TT in PwAD; (2) the standard error of measurement (SEM) and minimum detectable change (MDC) in the 360TT times; (3) the concurrent and known-groups validity of the 360TT times.

## Methods

### Study design

A cross-sectional study was conducted at the Department of Physiotherapy and Rehabilitation, Gazi University. This study received approval from the Gazi University Clinical Research Ethics Committee. Written informed consent was obtained from all participants before the procedures. Caregivers also provided informed consent on behalf of their care recipients. Data were collected between July and September 2025. The study was performed in accordance with the guidelines of the Declaration of Helsinki.

### Participants

The reliability of the 360TT has not been investigated in PwAD. The sample size calculation, thus, was based on a previous study of test-retest reliability of the 360TT for stroke (ICC = 0.824–0.951) [11], and PD (ICC = 0.860–0.932) [12], and older adults (ICC = 0.92) [14]. Assuming the ICC of 360TT times for PwAD is 0.90, a sample size of 30 participants would be required to achieve 80% power to detect an ICC value of 0.90 at a significance level of 0.05. The sample size was calculated using G*Power software, version 3.1.9.7 (Franz Faul, University of Kiel, Kiel, Germany) [15].

A convenience sample of PwAD, diagnosed with AD according to the criteria of the National Institute of Neurological and Communicative Disorders and Stroke and the Alzheimer’s Disease and Related Disorders Associations (NINCDS-ADRDA) [16], was consecutively invited by the neurologist in this study. The inclusion criteria were (i) being at least 65 years of age, (ii) a Clinical Dementia Rating Scale (CDR) score of ≤ 2 [17], (iii) Mini Mental State Examination score of ≥ 10 [18, 19], (iv) capable of walking at least 10 m independently with or without an assistive device. The exclusion criteria were (i) any neurological diseases other than AD and (ii) any orthopedic, vestibular, and rheumatologic conditions that can affect balance and gait. Cognitively healthy controls were recruited from the same clinical center and underwent neurological and neuropsychological examination to exclude the presence of any cognitive disorder. The same exclusion criteria were applied to the age- and gender-matched healthy people who were at least 65 years old and had a MMSE score of at least 26 [18].

### Outcome measures

The 360TT is a performance-based measure designed to evaluate turning ability. The test requires minimal equipment, consisting only of a visible floor marker (e.g., tape or pen) to indicate the starting point and a stopwatch or chronograph to measure performance. Participants stand comfortably at the marker in their usual walking shoes and are instructed to perform a complete 360° turn. The evaluator begins timing on the verbal command “go” and stops once the participant’s shoulders return to the initial forward-facing direction. The total time to complete the turn is considered as an indicator of turning performance [13]. Participants are instructed to turn at a self-selected, comfortable speed without the use of walking aids. One practice trial is allowed prior to formal testing and then three trials are performed in each direction. The mean value of the three trials for each direction is calculated and used for analysis [11].

The BBS is used to assess functional balance in 14 daily life activities. Each item is scored from 0 to 4 points with a total of 56 points. Higher scores reflect better balance ability [9].

The TUG evaluates functional mobility. Individuals rise from a chair with armrests, walk a distance of 3 m, turn around, return to the chair, and sit down. The time to complete the task is recorded in seconds using a stopwatch. Lower completion times indicate higher functional mobility performance [10].

The Mini-Mental State Examination (MMSE) is a brief and simple tool to measure global cognitive function. It is composed of 5 cognitive domains including orientation, registration, attention and calculation, recall, and language. The MMSE is scored from 0 to 30, with higher scores representing better cognition [18].

### Procedures

Demographic data collection and MMSE examination were performed by a neurologist. Then, motor assessments were administered in the following order: (1) 360TT, (2) BBS, and (3) TUG by the same physiotherapist in neurological physical therapy. One minute of rest period was given between assessments to avoid fatigue effects. To examine the test-retest reliability of the 360TT, PwAD were evaluated with the test twice with a 7–10 days interval. All conditions were kept as stable as possible; all assessments were evaluated at the same location, and around the same time of day.

The demographic data and MMSE score of healthy people were recorded by a neurologist. Subsequently, the 360TT was performed by the same physiotherapist in one assessment session. Their completion times of the 360TT were used to compare with those of PwAD.

### Statistical analyses

All data were analyzed using SPSS version 17 software (SPSS Inc, Chicago, Illinois). The Shapiro-Wilk test and Levene test were used to verify the normality of the data and homogeneity of variances. Descriptive statistics were expressed as mean ± SD for normally distributed data, median (interquartile range) for non-normally distributed data, and number (percentage) for categorical data. For between-group comparisons, the independent *t*-test was used for continuous variables with normal distribution, the Mann–Whitney U test was used for continuous variables with non-normal distribution, and the chi-square test was used for categorical variables.

The test-retest reliability was examined by repeating the same test twice over a period of time. The Shrout and Fleiss Type (2,1) ICC model was used to determine the test-retest reliability of the 360TT. Since the same physiotherapist scored all tests, a two-way random-effects single-measure model reliability analysis was calculated. For ICC values, test-retest reliability was categorized as follows: <0.40 poor, 0.40 ≤ ICC < 0.75 moderate, and ≥ 0.75 excellent [20]. Bland-Altman plot was built to display the agreement between the test and retest of the 360TT. The difference between the calculations was presented by plotting the mean bias and the 95% limits of agreement [21]. Measurement error represents the change in the measurement due to random and systematic errors unassociated with real changes in the constructs evaluated by the outcome measurement such as turning ability. Measurement error was assessed by the standard error of measurement (SEM) and the minimal detectable change (MDC). They were presented in the units of outcome measure (continuous data were recorded in seconds in here). SEM at the 95% confidence interval (SEM_95_) indicates the accuracy of the measurement tool, calculated by the following formula [22]:

$$SEM_{95}\:=\:SD_{pooled}\;x\;\surd(1-ICC)$$

MDC is defined as a real change score that is not due to chance. The MDC at the 95% confidence interval (MDC_95_) was determined according to the following formula [22]:

$$MDC_{95}\:=\:1.96\;x\;\surd2\;x\;SEM$$

This study determined both concurrent and known-groups validity of the 360TT for PwAD. First, the concurrent validity was assessed by computing the correlations of the 360TT with the other outcome measures using Pearson’s correlation coefficient (r). The strength of the correlations were classified as negligible (< 0.30), weak (0.30–0.49), moderate (0.50–0.69), strong (0.70–0.89), and very strong (0.90–1.00) [23]. Second, the known-groups validity was determined to compare the completion times of the 360TT between PwAD and healthy people [23]. Statistical significance was considered at *p* < 0.05 for all analyses.

## Results

Thirty-three PwAD (14 female, 19 male) and 32 healthy people (12 female, 20 male) were recruited for the study. Participants’ characteristics are presented in Table 1. There were no significant differences between the groups according to any demographic variables. However, PwAD had lower MMSE scores than healthy people (*p* < 0.001). The mean disease duration among PwAD was 4.93 ± 2.42 years, 23 of whom had a CDR score of 1, while the remaining 10 had a CDR score of 2. None of the participants required a walking aid in the assessment period.

**Table 1:** Participants’ characteristics

Characteristics	People with AD (*n* = 33)	Healthy people (*n* = 32)	*p*
Age, y Mean ± SD	74.42 ± 6.49	73.16 ± 4.18	0.355
Sex, n (%) Female Male	14 (42.4%) 19 (57.6%)	12 (37.5%) 20 (62.5%)	0.685
Height, cm Mean ± SD	170.84 ± 7.38	169.31 ± 8.85	0.450
Weight, kg Mean ± SD	74.66 ± 11.03	71.87 ± 8.03	0.249
BMI, kg/m^2^ Mean ± SD	25.51 ± 2.89	25.14 ± 2.86	0.604
Dominant side, n (%) Right Left	31 (93.9%) 2 (6.1%)	31 (96.9%) 1 (3.1%)	0.685
Education, y Mean ± SD	7.78 ± 3.31	8.65 ± 3.69	0.322
Disease duration, y Mean ± SD	4.93 ± 2.43	NA	NA
CDR score Median (IQR)	1.0 (1.00–2.00)	NA	NA
MMSE score Median (IQR)	22.00 (20.00–23.50.00.50)	28.0 (27.25–29.00.25.00)	< 0.001

AD, Alzheimer’s disease; BMI, body mass index; CDR, Clinical Dementia Rating Scale; cm, centimeter; IQR, interquartile range; kg, kilogram; kg/m^2^, kilogram/meter^2^; MMSE, Mini-Mental State Examinantion; NA, not applicable; SD, standard deviation; y, years

**Fig. 1:**
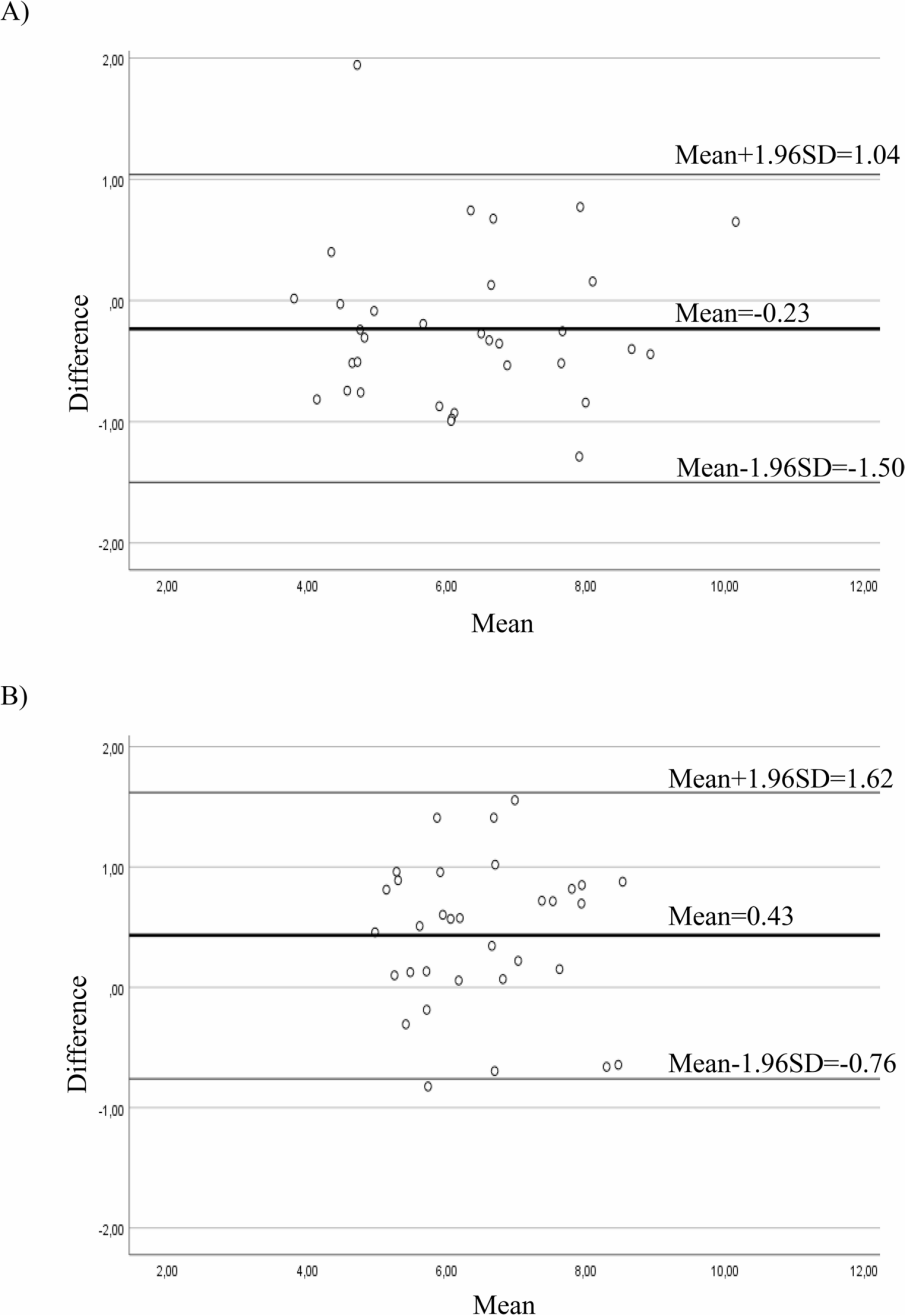
Bland–Altman plots for the test-retest reliability in people with Alzheimer’s Disease. (**A**) The 360º turn test times for the dominant side. (**B**) The 360º turn test times for the non-dominant side. The thick lines indicate the mean difference, and the thin lines indicates ± 1.96 standard deviation

Table 2 presents the test-retest reliability results of the timed 360TT for PwAD. For the 360TT, excellent test-retest reliability was found for the dominant and non-dominant sides (ICC = 0.957 and ICC = 0.916, respectively). The SEM_95_ and MDC_95_ values were 0.33 s and 0.91 s for the dominant side, while the SEM_95_ and MDC_95_ values were 0.31 s and 0.85 s for the non-dominant side. The Bland-Altman analysis displayed the mean absolute difference of −0.23 s with limits of agreement of 1.04 s (mean + 1.96SD) and − 1.50 s (mean-1.96SD) for the dominant side, while the mean absolute difference of −0.43 s with limits of agreement of 1.62 s (mean + 1.96SD) and − 0.76 s (mean-1.96SD) for the non-dominant side. Based on the Bland–Altman plots, only 1 data point was outside of the 95% limits of agreement for both sides. This demonstrated good agreement between the first and second assessments of the 360TT. Bland-Altman plots for the dominant and non-dominant sides are shown in Fig. 1.

**Table 2:** The test-retest reliability of the timed 360º turn test people with Alzheimer’s Disease

360TT	1st AssessmentMean±SD	2st AssessmentMean±SD	ICC(95% CI)	SEM_95_	MDC_95_
Dominant side	6.12±1.63	6.35±1.62	0.957 (0.913-0.979)	0.33	0.91
Non-dominant side	6.72±1.09	6.29±1.11	0.916 (0.829-0.958)	0.31	0.85

360TT, timed 360º turn test; CI, confidence interval; ICC, intraclass correlation coefficient; MDC, minimum detectable change; SD, standard deviation; SEM, standard error of measurement

The mean values of the other outcome measures and their correlations with the 360TT are presented in Table 3. The 360TT showed a strong positive correlation with the TUG (*r* = 0.774, *p* < 0.001), and moderate negative correlations with the BBS and MMSE (*r*=−0.545, *p* = 0.001 and (*r*=−0.527, *p* = 0.002)) for the dominant side, while it showed a moderate positive correlation with the TUG (*r* = 0.663, *p* < 0.001), moderate negative correlation with the BBS (*r*=−0.511, *p* = 0.002), and weak negative correlation with the MMSE (*r*=−0.363, *p* = 0.038) for the non-dominant side.

**Table 3:** Concurrent validity for the timed 360º turn test

Variables	People with AD (*n* = 33)	Dominant side		Non-dominant side	
		*r*	*p*	*r*	*p*
BBS score Mean ± SD	48.90 ± 5.11	−0.545	0.001	−0.511	0.002
TUG, sn Mean ± SD	16.74 ± 4.08	0.774	< 0.001	0.663	< 0.001
MMSE score Mean ± SD	21.39 ± 2.64	−0.527	0.002	−0.363	0.038

AD, Alzheimer’s disease; BBS, Berg Balance Scale; MMSE, Mini-Mental State Examination; r, Pearson’s correlation coefficient; SD, standard deviation; TUG, timed up and go test

There were significant differences in the completion times of the 360TT between PwAD and healthy controls for the dominant and non-dominant sides (*p <* 0.001 and *p <* 0.001, respectively) (Table 4).

**Table 4:** Known-groups validity of the timed 360º turn test

360TT	People with AD (*n* = 33)	Healthy people (*n* = 32)	*p*
Dominant side, s Mean ± SD	6.12 ± 1.63	3.36 ± 1.02	< 0.001
Non-dominant side, s Mean ± SD	6.72 ± 1.09	3.67 ± 0.93	< 0.001

360TT, timed 360º turn test; AD, Alzheimer’s Disease; s, seconds; SD standard deviation

## Discussion

This was the first study to investigate the reliability of the 360TT performed by PwAD. The results of this study showed excellent test-retest reliability, as demonstrated by the high ICC values and low SEM and MDC values. The correlations between the 360TT and AD-specific impairments such as functional balance, functional mobility, and cognitive status indicated acceptable concurrent validity, while higher completion times of PwAD on the 360TT of healthy people represented good known-groups validity.

An excellent test–retest reliability of the 360 TT was found for both sides in PwAD, which is consistent with that reported in stroke [11], PD [12], and older adults [14]. The 360TT provides consistent completion times when PwAD are assessed under the same conditions in two separate sessions. This excellent reliability contributes to clinical practice by enabling the evaluation of turning ability, which is a common and challenging daily living activity for PwAD. The excellent test-retest reliability of the 360TT in the AD was also supported by a very high agreement between occasions presented in the Bland–Altman plots.

ICC indicates the ability of measurement tool to detect differences between subjects despite measurement errors but cannot be generalizable to all other samples because of dependency on sample heterogeneity. Since ICC is not enough to provide a full picture of the reliability of a test, it should be used together with statistical indices expressing the measurement error of the test in a clinical practice. The SEM is an index that expresses a measure of stability over time [24]. The SEM for the 360TT score was less than half of the SD_pooled_ for the dominant and non-dominant sides (0.33 vs. 1.62 and 0.31 vs. 1.10, respectively), demonstrating good precision [25]. The SEM presents an error measurement value, which allows us to determine the amount of detectable change and the calculation of the MDC for the 360TT, while MDC is an index which expresses how much a test should change to provide confident information that it demonstrates a real change [22]. Our results indicate that, in individuals with AD, changes of ≥ 0.91 s for the dominant side and ≥ 0.85 s for the nondominant side on the 360TT represent real change and are not due to measurement errors. Clinicians should detect a change of this magnitude to be confident that turning ability reflects a true change.

The 360TT was correlated with the BBS. It can be considered that lower turning performance was related to an increased incidence of falls in neurological disorders such as PD [26] and stroke [27], which can reflect the link between turning and balance. The relative movement of body segments had an important role in controlling the center of mass within the base of support during functional activities [28, 29]. This can indicate that deterioration of the normal sequence of movement of body segments during turning could impact balance and explain why people with neurological disorders generally fall during turning. Such individuals are faced with a challenge during turning that aims to transport the body’s mass in a new direction. Turning requires deceleration of the body’s center of mass, rotation of the axial segments, and acceleration of the center of mass in the new direction [30, 31]. The requirements of turning can create unique challenges for individuals with impaired balance such as PwAD [32] because they are required to create a state of disequilibrium during single limb stance to change directions during an ongoing movement [31, 33]. Moreover, the mediolateral control that is crucial for maintaining balance during turns [34] was decreased in AD [35]. Therefore, turning may require adequate functional balance during daily living activities in PwAD.

There was a relationship between the 360TT and TUG. Turning performance was related to the functional mobility for stroke [11], PD [12], and older adults [36]. Turning speed was found to be a more responsive metric of age-related decline in mobility in terms of gait speed [37]. Turning, embedded within locomotion, plays a crucial role in functional mobility and is regularly required in daily life. Individuals often engage in functional tasks that involve turning and changing direction while walking [6]. For example, turning in various angles was performed to achieve both a basic household activity such as moving around furniture at home and walking in a crowded area outdoors. Additionally, turning is commonly evaluated through the TUG test [10]. Thus, the comprehensive functional mobility assessment for PwAD may include the 360TT as a more specific tool assessing turning performance.

A moderate correlation was found between the results of the 360TT and MMSE. The 180º turn performance was associated with the cognitive performance for PwAD during the TUG test [38]. Turning performance was associated with the processing speed and executive function [39], attention [40], perceptual speed, and visuospatial performance [41]. Moreover, turning requires higher demands on visual processing to achieve control of directional movement [42]. Deficits in balance [43] and gait [44] which are correlated with cognitive decline, can both contribute to turning dysfunctions. Motor and cognitive impairments generally occur through cognitive-motor interference. Thus, balance and gait can play a mediating role in the link between turning and cognition. This might suggest that the physical assessments particularly related to mobility impairments such as a turning ability should be added to the complementary assessments of cognitive status in the AD.

PwAD demonstrated worse performance than healthy controls to complete the 360° turn. Particularly, the loss of ability in turning [45], step quick turn-sway velocity and step quick turn-time [46] was reported in AD. Physical function gradually declines in the course of the disease [47]. This deterioration was indicated with the clinical and computerized physical performance tests measuring balance, mobility, postural stability, sensory organization, gait speed, step length [45, 46], step width, limits of stability, muscle strength, and reaction time [45], which probably impacted the turning performance of PwAD. Executive functions such as planning, attention, and sequencing can also be crucial for turning because individuals need to plan and execute turning without losing their balance [48, 49]. Since several factors can play a role in the loss of turning ability in AD, further studies are needed to clarify the underlying mechanisms responsible for turning performance.

## Limitations

There were some limitations in this study. All PwAD were community dwelling people and were at mild to moderate stages of the disease, which can restrict the generalization of the findings. Future studies are needed to validate this test in PwAD who are institutionalized and at the late stages of the disease. This study only evaluated the completion the 360TT but did not assess other variables such as step, symmetry, muscle strength, and proprioception. Prospective studies should focus on these parameters which impact turning performance while performing the 360TT. Another limitation was that the causality between turning performance and functional balance, functional mobility, and cognitive function was not investigated in this study. Therefore, longitudinal studies should be designed to establish a causal relationship between turning ability and disease-specific impairments for PwAD.

## Conclusions

The excellent test-retest reliability of the 360TT indicated that it may be an applicable standardised method to assess turning performance in AD. The MDC values suggested that a change of at least 0.91 s for the dominant side and 0.85 for the non-dominant side is required to represent better or worse performance of the 360TT. Turning ability measured with the 360TT was related to functional balance, functional mobility, and cognitive status. PwAD had worse turning performance compared to healthy people, indicating that the 360TT can detect turning deficits. In conclusion, the 360TT may be a reliable and valid performance-based test to measure turning ability for PwAD. Therefore, the 360TT can be usefully integrated into the clinical practice of AD.

## Data Availability

All data generated or analyzed during this study are included in this published article.
